# Incidence of catheter-related bloodstream infection (CRBSI) in immunosuppressed hosts post solid organ transplant (SOT): a single center experience

**DOI:** 10.3389/frtra.2025.1586035

**Published:** 2025-05-14

**Authors:** Christopher El Mouhayyar, Ayman Al Jurdi, Kassem Safa

**Affiliations:** ^1^ Division of Nephrology, Department of Medicine, Massachusetts General Hospital, Boston, MA, United States; ^2^ Harvard Medical School, Boston, MA, United States; ^3^ Center for Transplantation Sciences, Massachusetts General Hospital, Boston, MA, United States

**Keywords:** AKI, hemodialysis, organ transplant, CRBSI, TDC

## Abstract

**Introduction:**

Catheter-related bloodstream infections (CRBSI) incidence is well-studied in general hemodialysis patients. There is a lack of data on CRBSI rates specifically in solid organ transplant (SOT) recipients requiring hemodialysis. This study aims to investigate CRBSI incidence in this population at a single center.

**Methods:**

This retrospective, single-center cohort study at Massachusetts General Hospital (MGH) investigated CRBSI incidence in non-kidney SOT (i.e., heart, lung, liver) who required hemodialysis via a tunneled dialysis catheter (TDC). Data was collected from January 2016 to October 2024, with patients followed for up to two years post-transplant or until death/end of study.

**Results:**

42 individuals met the study's inclusion criteria. The mean age of this cohort was 57 years, 50% were male, and 81% were White. The group consisted of 17 liver transplant recipients (40.5%), 13 heart transplant recipients (31.0%), and 12 lung transplant recipients (28.6%). Among the 12 lung transplant recipients, 8 received basiliximab induction, and 4 received no antibody induction therapy. 97% of the patients received mycophenolate mofetil, tacrolimus, and prednisone, while 3% received steroid-free maintenance. The median follow-up was 51.5 days (interquartile range 16–233). During this period, six individuals developed CRBSI, resulting in an incidence rate of 0.86 infections per 1,000 catheter-days. No deaths were attributed to CRBSI.

**Conclusions:**

Our findings suggest that intense immunosuppression in the setting of SOT is not associated with an increased risk of CRBSI in patients with renal failure utilizing TDC especially when a consistent and standardized protocol for the access and care of these catheters is utilized.

## Introduction

In 2023, the United States performed a total of 46,629 organ transplants, with an anticipated increase in the number of transplants completed during 2024 (1); among those, 18,223 patients underwent liver, lung, or heart transplant. Dialysis-dependent kidney failure, whether acute or chronic is a well known complication of non-kidney (liver, lung, or heart) solid organ transplantation (SOT) (2–4). Furthermore, hemodialysis carries a significant risk of infection due to the patients' compromised immune systems and repeated access to the bloodstream (5, 6). Infections account for a substantial proportion of post-transplant mortality, ranging from 13% to 16% in kidney and heart recipients, up to 21% in lung recipients and approximately 50% within the first year after liver transplantation (7).

Among the various hemodialysis vascular access types, central venous catheters (CVCs) are associated with the highest infection risk (8, 9). Catheter-related bloodstream infection (CRBSI) is a serious complication associated with CVC use in patients on hemodialysis (10) and is associated with increased hospitalization and mortality rates. While the incidence rate of CRBSI in the general hemodialysis population is well-documented (8, 10–12), there are no published data on CRBSI incidence in solid organ transplant recipients with renal failure (acute or chronic) requiring hemodialysis and it is unknown if the intense immunosuppressed status post SOT is associated with an increase risk for CRBSI.

This study aims to address this knowledge gap by examining the incidence of CRBSI post-organ transplant at our center and comparing our findings to national rates.

## Materials and methods

### Study design

This retrospective observational single-center cohort study at Massachusetts General Hospital (MGH) examined the incidence of catheter-related bloodstream infections (CRBSI) in non-kidney solid organ transplant (SOT) recipients (heart, lung, liver) requiring intermittent hemodialysis or continuous renal replacement therapy via a tunneled dialysis catheter (TDC). The study period spanned January 1, 2016—October 31, 2024, a nearly 9 year duration, with patients followed for up to 2 years, death, or study end. The study adhered to STROBE guidelines and was approved by the Massachusetts General Brigham IRB (2025P000242) with a waiver of informed consent.

### Data source and study population

Patients were identified through EMR reports of TDC insertions in SOT recipients, with manual chart review confirming eligibility. Inclusion criteria: (1) age ≥18 years, (2) history of non-kidney SOT, and (3) renal failure requiring dialysis via TDC. We excluded kidney transplant recipients only but not silmutaneous liver and kidney (SLK) recipients where patients were not dialysis dependent prior to the SLK, to focus on non-renal SOT recipients, where the risks and management of renal failure requiring dialysis are distinct. Kidney transplant recipients typically have pre-established nephrology care and dialysis plans, unlike non-kidney recipients. Further, most kidney transplant patient who present for a kidney transplant have already established dialysis access and the dialysis needs after transplant are often limited to 1–2 weeks in the case of delayed kidney graft function. Data were entered into a secure web application.

### Data collection & outcomes

The primary outcome was CRBSI, defined as a bloodstream infection attributed to TDC use, confirmed by two nephrologists and an infectious disease consultant. Collected variables included demographics, comorbidities, transplant type, immunosuppression, and dialysis access details.

### MGH CVC access protocol

MGH Dialysis Nurses follow a strict protocol to prevent CRBSIs, using See-Luer caps and sterile techniques for accessing TDCs. The protocol includes:

• Before access: Hand hygiene, sterile gloves, and a sterile field. Ports are disinfected with alcohol pads (15 s), then See-Luer caps before flushing with saline.
• Post-session: Catheters are flushed with saline + anticoagulant, disinfected, and sealed with See-Luer caps. Used catheters are labeled appropriately.

### Statistical analysis

Continuous variables are summarized as mean (±SD) or median (IQR), categorical as counts (percentages). CRBSI incidence was calculated as total events per catheter-days, defined as TDC duration from placement to removal, death, or study end. Given the small sample size and descriptive nature of the data, we focused on incidence rates (per 1,000 catheter-days) rather than inferential statistics.

## Results

### Study population

During the study period from January 1st, 2016 to October 31st, 2024, 42 individuals met our study's inclusion. Table 1 shows the characteristics of the cohort. The mean age was 57 years, 50% were male, and 81% were White. The cohort included 17 liver transplant recipients (40.5%), 13 heart transplant recipients (31.0%), and 12 lung transplant recipients (28.6%).

**Table 1:** Characteristics of patients on dialysis after solid organ transplant.

Characteristics (*n* = 42)	Frequency (%)
Age (Mean +/− SD)	57.0 +/− 11.0 years
Gender (Male)	21 (50%)
BMI (Mean +/− SD)	28.0 +/− 5.6
Race/Ethnicity	
White	34 (81%)
Black	3 (7%)
Hispanic	3 (7%)
Asian	2 (5%)
Comorbidities	
Diabetes mellitus	9 (21%)
Hypertension	13 (31%)
Coronary artery disease	12 (29%)
Transplanted organ	
Liver	17 (40.5%)
Heart	13 (31.0%)
Lung	12 (28.6%)
Organism speciation	
Enterococcus Faecalis	3/6 (50%)
Candida Krusei	1/6 (16.7%)
Prevotella oris	1/6 (16.7%)
Enterococcus Faecium	1/6 (16.7%)
Induction Immunosuppression:	
No antibody induction	34 (81%)
Basiliximab	8 (19%)
Maintenance Immunosuppression:	
Mycophenolate mofetil, tacrolimus and prednisone	41 (97%)
Tacrolimus and prednisone	1 (3%)

### Immunosuppressive regimen

None of the heart or liver transplant recipients received antibody induction therapy. Among the 12 lung transplant recipients, 8 received basiliximab induction and 4 received no antibody induction therapy. With regards to maintenance immunosuppression 97% of the patients received maintenance immunosuppression with mycophenolate mofeti, tacrolimus and prednisone, while 3% received steroid-free maintenance. 5 patients experienced transplant rejection. 2 of these were liver transplant recipients, both of whom were treated with steroid pulse therapy. The remaining 3 patients were lung transplant recipients; 1 of these received basiliximab, and the other 2 received steroid pulse therapy.

### Acute kidney injury

Among the 42 individuals meeting inclusion criteria, identified causes of renal dysfunction included ischemic acute tubular necrosis (i-ATN) in 21 individuals (50%), per nephrology consultation, hepatorenal syndrome in 14 (34%), cardiorenal syndrome in 5 (12%), delayed graft function (DGF) in 1 (2%), and calcineurin inhibitor-associated acute kidney injury (CNI-AKI) in 1 (2%). At the time of dialysis initiation, all transplanted organs (heart, liver, or lung) were functional and renal failure was not due to primary graft dysfunction.

### Incidence rate of CRBSI

The median follow-up was 51.5 days (IQR 16–233). During follow-up, six individuals developed CRBSI. The incidence rate of CRBSIs in the cohort was 0.86 infections per 1,000 catheter-days. There were no deaths due to CRBSI.

### CRBSI microbiology

**Figure 1:**
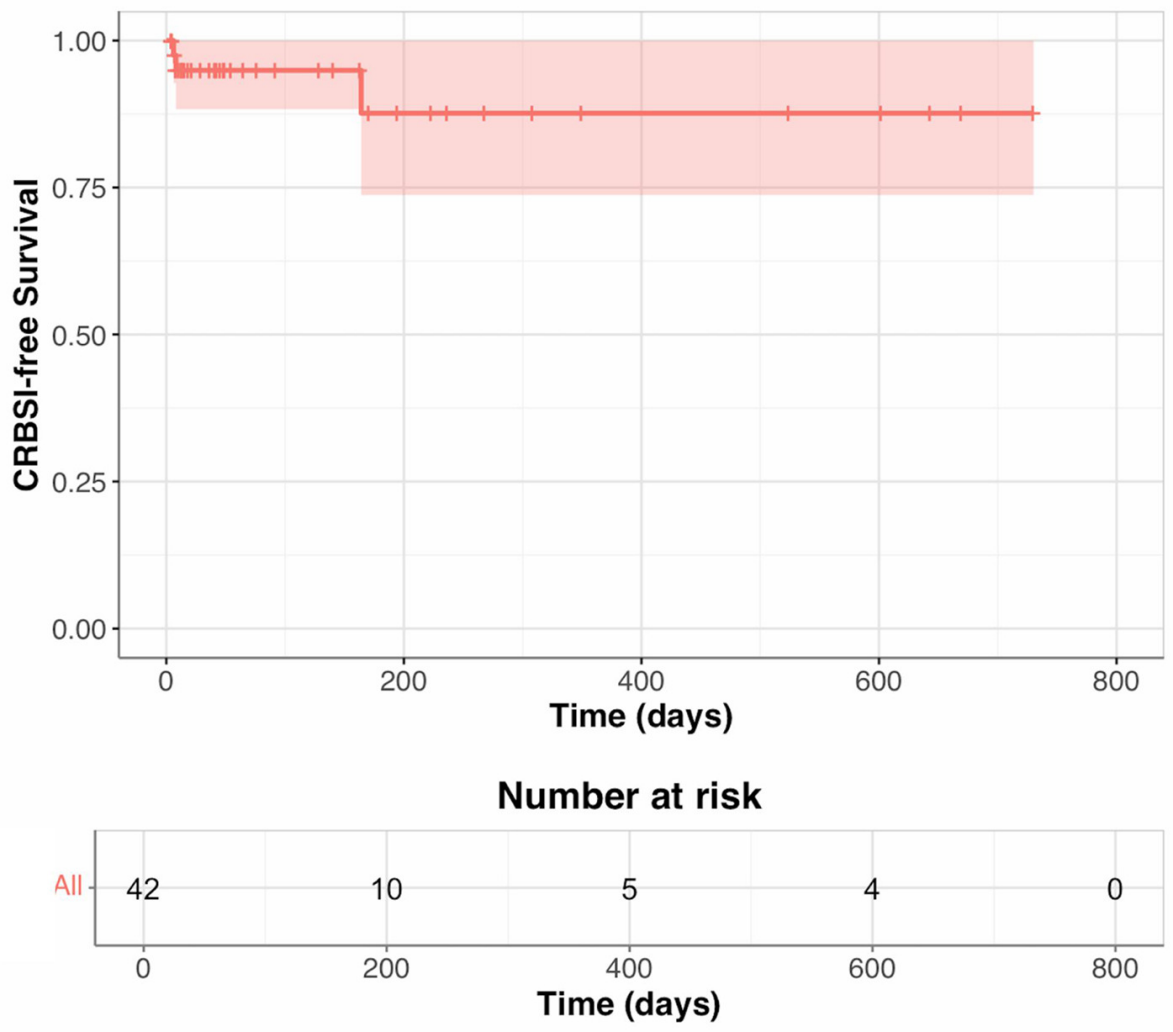
CRBSI-free survival by Kaplan–Meier method.

During the first 90 days of follow-up, two patients developed CRBSIs: one by *Candida krusei* and the other by *Prevotella oris*. Between 90 and 179 days, there was one CRBSI due to *Enterococcus faecalis*. Between 180 and 365 days, three CRBSIs occurred: two *Enterococcus faecalis* infections and one *Enterococcus faecium* infection. From days 366 to 730, no CRBSIs occurred (Table 2; Figure 1).

**Table 2:** Causes of renal failure, acute rejection episodes, and graft function at dialysis initiation.

Characteristics (*n* = 42)	Frequency (%)
Causes of renal failure	
Ischemic acute tubular necrosis (i-ATN)	21 (50%)
Hepatorenal syndrome	14 (34%)
Cardiorenal syndrome	5 (12%)
Delayed graft function (DGF)	1 (2%)
Calcineurin inhibitor-associated acute kidney injury (CNI-AKI)	1 (2%)
Graft Rejection	
Liver	2 (5%)
Heart	0
Lung	3 (7%)
Anti-rejection treatment	
Steroids	4 (10%)
Basiliximab	1 (2%)
Graft function at dialysis initiation	
Functional	42 (100%)
Non-functional	0

## Discussion

CVC use is prevalent in US ICUs, with an estimated 15 million days of CVC exposure reported annually, which includes the total number of days CVC exposure among all patients in a selected population over a time period (13). CRBSIs are a significant concern, independently increasing hospital costs and length of stay (14, 15). Mortality rates associated with CRBSIs have declined, from 7.9% in 1997 to 5.9% in 2008 according to the National Hospital Discharge Survey (16). However, a 10-year cohort study reported 17.4% of patients with CRBSIs died within 30 days, with a population attributable mortality rate of 18.2% (17). The overall cost of these infections remains substantial, both in terms of morbidity and resource utilization.

This is the first report to describe the incidence rate of CRBSI in non-kidney solid organ transplant recipients, an intensely immunosuppressed population. CRBSIs are a significant concern for patients with tunneled dialysis catheters (TDCs). The incidence rate of CRBSIs varies widely in studies of the general hemodialysis population, ranging from 0.5 to 5.5 episodes per 1,000 catheter-days, likely influenced by factors such as patient population, catheter care practices, and duration of catheter use (12). Higher rates are typically observed in settings with suboptimal infection control protocols or among patient groups with greater comorbidities. Several risk factors have been identified, including prolonged catheter dwell time, underlying conditions such as diabetes mellitus and immunosuppression, and inadequate catheter care practices (18). The Centers for Disease Control and Prevention (CDC) recommend maintaining CRBSI rates below 1.0 episode per 1,000 catheter-days in facilities adhering to best practices (19). However, achieving these benchmarks often depends on the implementation of strict aseptic techniques, regular staff education, and preventive measurements.

In our cohort of intensely immunosuppressed patients after solid organ transplantation, the incidence rate was 0.86 infections per 1,000 catheter-days. This rate is comparable to CRBSI rates reported in general hemodialysis patients with TDCs who are not SOT recipients. Our findings suggest that intense immunosuppression in the setting of solid organ transplantation is not associated with an increased risk of CRBSI in patients with renal failure utilizing TDCs especially when a consistent and standardized protocol for the access and care of these catheters is utilized.

The study provides needed insight about the safety of TDCs in immunosuppressed hosts with renal failure. Recent solid organ transplant recipients with acute kidney injury requiring dialysis are often not readily amenable for dialysis access surgery or kidney transplants and as such TDCs are a needed bridge. However, there are important limitations to consider. First, the retrospective cohort observational design only allows us to make associations and is susceptible to confounding factors. Second, the single-center design, the moderate sample size, and the inability to run inferential statistitics may affect the generalizability of our estimate for the CRBSI incidence rate. Lastly, the comparison of incidence rates between studies depends on the assumption of person-time equivalence, which assumes that the risk of the outcome during any person-time period is equivalent to the risk during any other person-time period, and that assumption may or may not be true during follow-up. This limitation could be overcome in the future, utilizing post transplant national registries mandating the reporting of this specific complication in SOT recipients.

## Data Availability

The raw data supporting the conclusions of this article will be made available by the authors, without undue reservation.
